# Genome wide identification and analysis of inositol tetrakisphosphate kinase (*ITPK*) gene family in *Triticum aestivum* L.

**DOI:** 10.1371/journal.pone.0359528

**Published:** 2026-09-29

**Authors:** Fatima Umar, Rizwana Maqbool, Muhammad Ahsan Khan, Bushra Sadia

**Affiliations:** 1 Department of Plant Breeding and Genetics, Faculty of Agriculture, University of Agriculture, Faisalabad, Pakistan; 2 Centre of Advanced Studies in Agriculture and Food Security, University of Agriculture, Faisalabad, Pakistan; 3 Centre of Agricultural Biochemistry and Biotechnology, University of Agriculture, Faisalabad, Pakistan; Institute of Genetics and Developmental Biology Chinese Academy of Sciences, CHINA

## Abstract

Inositol tetrakisphosphates kinase (*ITPK*) plays a vital role in regulating cellular responses to abiotic stress, plant hormone signaling and various other aspects of plant growth and development. Additionally, it is also involved in phosphorous, inositol phosphate metabolism and synthesize phytic acid which is the main phosphorous storage form in seeds. While essential for plant physiology, high phytic acid levels reduce the bioavailability of minerals such as Fe and Zn, which impact nutritional quality negatively. Despite its importance, the *ITPK* gene family remains largely unexplored in bread wheat. In this study, we conducted a comprehensive genome-wide analysis, including phylogenetic analysis and expression profiling, of the *ITPK* gene family in wheat. We systematically examined gene structure, chromosomal distribution, motif and domain conservation, promoter analysis, synteny analysis, protein modeling and protein-protein interaction of *ITPKs*. A total of 15 *ITPK* genes have been identified in wheat (*T. aestivum*). The *TaITPK* genes were distributed on chromosome number 1, 4, 5 and 7 in all the three genomes A, B and D. Comparative phylogenetic analysis resolved four major groups, with wheat *ITPK* sequences represented in Groups I, III, and IV, whereas Group II contained no wheat sequence. The conserved domain and motif analysis showed the presence of Ins134_P3_kin domain at N-terminus and CPase_L_D2 in C-terminus of protein. String based protein interaction analysis suggested *ITPK* genes interact with Inositol polyphosphate multi kinase (*IPK1*) and Inositol-pentakisphosphate 2 kinase (*IPK2*) which were also involve in synthesis of phyti acid. Furthermore, in-silico expression analysis of *ITPK* genes revealed their preferential tissue-specific and growth stage expression, especially after anthesis of wheat. Preliminary qRT-PCR profiling of selected *TaITPK* genes revealed treatment-dependent relative expression patterns under FeSO_4_ and ZnSO_4_ supplementation. The current study has identified 15 *ITPK* genes present in hexaploid wheat genome. Their evolutionary, structural, and expression analyses provide a foundation for further functional characterization of this gene family in wheat.

## 1. Introduction

Phytic acid (PA; myo-inositol hexakisphosphate, IP6) is abundantly present plant compound that constitutes about 1–5% of the dry weight of edible legumes, cereals, oilseeds, and nuts which are the major components of human and animal diets [1]. The phytate content in these foods varies greatly, depending on several factors such as crop species, soil type, growing conditions, fertilizer application, harvesting time, post-harvest processing, and analytical methods used for quantification [2]. Generally, foods cultivated with modern high-phosphate fertilizers tend to accumulate higher levels of phytic acid compared to those grown using organic or compost-based systems [3]. Due to its highly phosphorylated inositol ring, PA readily forms insoluble complexes with multivalent cations [4,5].

In plants PA is synthesized through two pathways: lipid-dependent and lipid-independent [6]. In cereal and legume seeds, the lipid-independent pathway follows which begin with the conversion of glucose-6-phosphate to inositol-3-phosphate by myo-inositol-3-phosphate synthase (MIPS) [7]. Successive phosphorylation by various kinases then produces inositol pentakisphosphate (IP5), which is finally converted into phytic acid (IP6) [8]. The phosphorylation cascade leading from inositol triphosphate (IP_4_) to inositol hexakisphosphate (IP₆) represents one of the most conserved biochemical pathways in eukaryotes [9]. Among extensive research into inositol phosphate metabolic enzymes in plants, the Inositol teris/tetrakisphosphate kinase (ITPK) also known as Inositol-1,3,4-triskisphosphate 5/6-kinase (ITP5/6 K) gene family receives special attention because it is abundantly present in plants and catalyzes the penultimate step of Phytic acid synthesis [10]. It plays a crucial intermediary role by catalyzing the conversion of inositol trisphosphate (IP_3_) to inositol pentaphosphate (IP_5_) via sequential phosphorylation [11]. Through this role, ITPKs not only contribute to phytic acid biosynthesis, but also participate in energy metabolism, phosphate signaling, and abiotic stress responses. Since late 1980s, numerous studies have also demonstrated its beneficial roles, highlighting its antioxidant, anti-carcinogenic, and antidiabetic properties [12,13].

However, Phytic acid has been known as an anti-nutrient agent since long [14–16]. In plants, phytic acid primarily occurs as salts of mono- and divalent cations such as K ⁺ , Mg² ⁺ , and Ca² ⁺ , and it accumulates in seeds during the maturation phase [17–19]. Monogastric animals, including humans and poultry, cannot effectively metabolize phytic acid because their digestive systems lack adequate phytase enzyme activity required to hydrolyze phytate and release its bound nutrients [20]. This property is of great concern in cereal-based diets, particularly in developing countries where wheat constitutes a primary food source. Chronic deficiency of Fe and Zn leads to hidden hunger, a form of malnutrition affecting billions of people worldwide [21]. Therefore, a major goal of modern wheat improvement programs is to reduce phytate content while maintaining adequate phosphorus storage for seed viability.

Since *ITPKs* catalyze the rate-limiting steps in the conversion of lower inositol phosphates to phytic acid, understanding their gene structure, expression regulation, and evolutionary conservation is an essential step toward developing low-phytate, micronutrient-rich wheat varieties. However, the understanding of *ITPKs* for its functional role and genetic diversity in wheat not studied extensively, especially given the complex polyploid nature of the wheat genome. In recent years, significant interest has been directed towards the *ITPK* family of enzymes in different crops due to their role in the sequential phosphorylation steps involved in phytate synthesis in cereal crops like maize and rice but limited in wheat. In wheat, the expression pattern of the selected genes involved in the final step of biosynthetic pathway of phytic acid at different growth stages has been studied which suggest their role in phytic acid accumulation. However, their response to iron and zinc availability is not well understood. Recent advances in bioinformatics tools have made it possible to conduct comprehensive genome-wide studies, enabling the identification of gene families across complex polyploid genomes.

The present study was undertaken to perform a genome-wide identification analysis of the *ITPK* gene family in *Triticum aestivum* L. First, *TaITPK* gene members were identified and their chromosomal distribution was determined. Subsequently, their phylogenetic relationships, syntenic associations, and protein structural features were analyzed to understand their evolutionary origin and diversification. To further explore their potential regulatory mechanisms, promoter regions were examined for cis-regulatory elements. Finally, expression profiling under different iron and zinc conditions was carried out to assess the possible functional roles of *TaITPK* genes in wheat. This will provide a strong basis for understanding *ITPK*s family members in wheat.

## 2. Materials and methods

### 2.1. Identification, classification and phylogenetic tree construction of ITPK gene family

The protein sequence of *Arabidopsis Thaliana* (AT5G16760, AT4G33770, AT4G08170, AT2G43980) and *Oryza sativa* (LOC4347597) *ITPK* gene family were used as query retrieved from TAIR and NCBI. The query proteins were BLASTP to search against *Triticum aestivum* in Ensembl Plant https://plants.ensembl.org/index.html to find out wheat homeologs with cut off E-value < 1e^−5^. The candidate proteins were further screened out using NCBI CD search https://www.ncbi.nlm.nih.gov/Structure/bwrpsb/bwrpsb.cgi. Moreover, molecular weight, theoretical iso electrical point Pi, GRAVY, No. of amino acids were calculated using Expasy ProtParam https://web.expasy.org/protparam/. The chromosome ID and gene location were retrieved from Ensembl Plant https://plants.ensembl.org/index.html. The subcellular localization of each protein was predicted using CELLO: Subcellular Localization Predictive system database http://cello.life.nctu.edu.tw/

Furthermore, protein sequences of *Arabidopsis thaliana*, *Zea mays*, *Hordeum vulgare* and *Oryza sativa* were selected along with *Triticum aestivum* as these species represented well-characterized dicot and monocot plants and facilitate evaluation of the evolutionary relationships, conservation, and divergence of the *ITPK* gene family. These protein sequences were aligned in MUSCLE https://www.ebi.ac.uk/jdispatcher/msa/muscle?stype=protein with default parameters using MEGA 11 and IQ-TREE was used to construct an maximum likelihood (ML) phylogenetic tree with 1000 bootstrap repeats for ultra-fast bootstrapping [22]. The phylogenetic tree was visualized using iTOL v7 (https://itol.embl.de (accessed on 7 August 2025)) [23].

### 2.2. Gene structure, conserved motif and domain analysis

To analyze the conserved motifs present in the wheat *ITPK* gene family, the MEME Suite web server https://meme-suite.org/meme/ was used providing the full-length protein sequences, with the maximum number of motifs set to ten [24]. The exon–intron organization of *ITPK* genes was examined using GSDS 2.0 https://gsds.gao-lab.org/Gsds_help.php which provided a graphical representation of gene structure. Domain analysis was performed using the NCBI Conserved Domain Database (CD-Search) tool to identify and confirm functional protein domains. Finally, an integrated visualization combining gene structure, conserved motifs, and domain architecture was generated using the Gene Structure View (Advanced) function in TBtools-II [25].

### 2.3. Comparative synteny analysis of *ITPK* genes

To further investigate the conservation and evolutionary relationships of *ITPK* genes, a synteny analysis was performed between *Triticum aestivum* and other plant genomes (*Oryza sativa*, *Zea mays*, *Hordeum vulgare*, and *Arabidopsis thaliana*). The genomic sequence (FASTA) and genomic annotation (GFF3) files were retrieved from the Ensembl Plants database. Homologous gene pairs were identified using protein sequence with an E-value threshold of 1e − 10, and the resulting alignment file, along with the GFF annotation, was used as input for MCScanX. The syntenic relationships among *ITPK* genes were visualized in TBtools-II [26]. Conserved syntenic blocks containing *ITPK* genes were identified to trace evolutionary conservation.

### 2.4. Gene location on chromosomes and gene divergence analysis

The chromosomal distribution map of wheat *ITPK* genes was generated using the Gene Location Visualize (Advanced) function in TBtools-II. The chromosome lengths, physical positions, and length of each gene were retrieved from the Ensembl Plants and used to map the locations of *TaITPK* genes across wheat chromosomes. Furthermore, to estimate sequence divergence among *ITPK* paralogous gene pairs, the coding sequences (cds) were aligned using MUSCLE algorithm in MEGA11. The evolutionary divergence among paralogous pairs was calculated by using Nei-Gojobori method (Jukes-Cator) model [27]. After that, selection pressure was estimated with non-synonymous (Ka) and synonymous (Ks) substitution rates via Pairwise dN/dS estimation tool in MEGA11. The dN/dS (Ka/Ks) ratio was calculated to infer the type of selection acting on duplicated genes [28].

### 2.5. Protein modeling and Protein-protein interaction

The three-dimensional (3D) structures of *TaITPK* proteins were predicted using the SWISS-MODEL online server (https://swissmodel.expasy.org/) [29,30]. The peptide sequences of each *TaITPK* protein were submitted to the server, and homology-based structural models were generated. The reliability and quality of the predicted models were evaluated based on Global Model Quality Estimation (GMQE) and Qualitative Model Energy Analysis (QMEAN) scores [31,32].

Moreover, For protein-protein interaction prediction, STRING database was used (https://string-db.org/) by uploading the peptide sequences of *TaITPK* genes [33,34]. The interaction network was constructed to identify potential interacting partners and associations among *TaITPK* proteins.

### 2.6. Cis-regulatory element (Promoter) analysis

For analyzing the cis-acting regulatory element of each *TaITPK* gene, the 1.5 kb upstream region of each gene was retrieved from the Ensembl Plants database (*Triticum aestivum* genome, IWGSC RefSeq v2.1). The extracted promoter sequences were analyzed using the PlantCARE database (https://bioinformatics.psb.ugent.be/webtools/plantcare/html/) to identify putative cis-regulatory motifs [35]. Visualization of cis-element distribution was carried out using TBtools-II using the “Simple Bio-sequence Viewer” module [36].

### 2.7. In silico expression analysis of *TaITPK* genes

For in silico expression analysis, RNA seq data for expression analysis of *TaITPK* genes in different tissues at different growth stages and under drought and heat stress in wheat was downloaded from Wheat Exp (https://dubcovskylab.ucdavis.edu/wheatexp) [37]. Heat map showing expression of each gene was constructed in TBtool.

### 2.8. Plant material and application of FeSO_4_ and ZnSO_4_

Two wheat (*Triticum aestivum* L.) genotypes, ‘Akbar-2019’ and ‘Fateh Jang-2016’, were cultivated at the experimental field of the Department of Plant Breeding and Genetics, University of Agriculture, Faisalabad, during the 2024–2025 wheat growing season. The experiment consisted of four treatments, i.e., Control (no micronutrient application), Soil application of FeSO_4_ (20 kg ha^−1^), Soil application of ZnSO_4_ (20 kg ha^−1^), and combined FeSO_4_ + ZnSO_4_ application (each at 20 kg ha^−1^) [38]. The soil collected from 0−15 cm surface from the selected field have range of 1.002–1.012 ppm Zn and 1.614–1.695 ppm Fe. For RNA extraction, spikes were sampled 14 days after anthesis (DAA). The collected samples were immediately frozen in liquid nitrogen and stored at −80°C until further molecular analysis.

### 2.9. RNA isolation, cDNA synthesis and qRT-PCR analysis

RNA was isolated using TRIzol^TM^ Reagent [39]. Extracted RNA integrity was determined using 1% agarose gel electrophoresis and quantification of RNA through Nano-drop-8000. For the synthesis of cDNA, First Strand cDNA kit Thermo-Scientific Revert Aid (K1622; 100rxns) was used and protocol was followed according to manufacturer guidance [40]. The primers information is given in S5 Table. Then, the Thermo-Scientific Maxima SYBR green Master mix was used. The *Actin* (housekeeping gene) was used as internal reference gene for comparison with transcript of target genes. The list of primers used mentioned in S5 Table (See Supporting Information). The reaction mixture contained 0.5µl of each forward and reverse primer, SYBR green 6µl, cDNA 1µl and water 2µl. Total 10µl reaction was prepared. Then expression was determined using Quant Studio 5 real time PCR machine and following profile was used: Initial denaturation at 95°C for 10 min, denaturation at 95°C for 15 seconds and annealing of primer at 60°C for 1 minute and 40 cycles.

The relative expression levels were calculated using the 2^−△△𝐶𝑡^ method, *ΔCt = Ct_target − Ct_actin and ΔΔCt = ΔCt_treatment − ΔCt_control* [41]. For each genotype × treatment combination, qRT-PCR measurements were performed in three technical repeats from one biological sample. The relative expression values are presented as mean ± standard error (SE) of the technical measurements. As independent biological replicates were not available, no inferential statistical analysis was performed, and the expression patterns were interpreted descriptively.

## 3. Results

### 3.1. Identification, classification and Phylogenetic analysis of *ITPK* gene family

The protein sequence of *ITPK* genes were identified through BLASTP in *Triticum aestivum* using query sequence of *Arabidopsis Thaliana* and *Oryza sativa* https://plants.ensembl.org/index.html. After removing redundant, the remaining genes were further screened for conserved domain analysis in NCBI-CD Search https://www.ncbi.nlm.nih.gov/Structure/bwrpsb/bwrpsb.cgi and incomplete domains were removed. Furthermore, through Motif finder, pseudogenes with lack of inositol 1,3,4 trisphosphates 5/6 kinase were also excluded. The full length transcripts of all identified genes were aligned through ClustalW https://www.genome.jp/tools-bin/clustalw to check any differences among them. Our analysis yielded 15 *ITPK* genes of *Triticum aestivum*. The gene IDs, chromosome IDs, gene locus, no. of amino acids, molecular weight, isoelectric point and subcellular localization are listed in S1 Table. (*See Supplementary Information*). The no. of amino acids varied from 214 to 503. The GRAVY ranges from −0.098 to 1.56. The *TaITPKs* were predicted to localized in mitochondria, chloroplast, cytoplasm, extra cellular and plasma membrane. Most of the genes were present in cytoplasm. The average molecular weight and isoelectric point of *ITPK* gene family was 41.06 kda and 5.60 respectively and showed large variation.

We constructed the Maximum Likelihood Phylogenetic tree of *Triticum aestivum* with *Oryza sativa*, *Zea mays* and *Arabidopsis thaliana ITPK* proteins. The identified wheat *ITPK* genes were named with reference to previously reported homologous *ITPK* genes in related cereal species and were further distinguished according to their chromosomal locations and corresponding A, B, and D subgenomes. The numerical gene designations are independent of the phylogenetic Groups I–IV. The phylogenetic analysis classified the *ITPK* into four distinct clades reflecting their evolutionary divergence. The multiple homeologous present in wheat within this clade indicated a significant level of gene expansion, might be the result of polyploidization and tandem duplication. The close clustering of the sequences showed evolutionary conserved origins. In case of Group I, *ITPK6* sequences formed a distinct and relatively compact cluster. It’s separation from other clades identified early evolutionary divergence. Group 1 also included *AtITPK4* in it which showed more similarity to *OsITPK6*. Total three *ITPK6* genes are present in this clade. Group II did not contain any wheat sequence, whereas *AtITPK1, ZmITPK1*, and *OsITPK5* were present in this group. No corresponding wheat sequence was recovered under the identification criteria used in the present study. The absence of a wheat member may reflect lineage-specific gene loss or sufficient sequence divergence to prevent its detection under the present identification criteria. However, these possibilities cannot be conclusively distinguished based on the present phylogenetic analysis alone.

The Group III included *ITPK1* and *ITPK4* gene groups indicating a shared evolutionary origin. Despite being classified as different subgroups, phylogenetic association of *ITPK1* and ITPK4 showed that they may come from common ancestral gene. The *TaITPK1* homoeologs clustered most closely with the barley *HvITPK1* sequence in the present phylogenetic analysis. The *ITPK4* group of *T. aestivum* showed similarity with *H. vulgare*, *O. sativa* and *Z. mays* but not with *A. thaliana*. This clade also doesn’t include *AtITPK4* gene. Total six members of wheat included in Group III. The *T. aestivum ITPK2* genes showed similarity with *ITPK2* genes of *H. vulgare*, *O. sativa* and *Z. mays*. The *OsITPK1* gene also showed similarity with *ITPK2* genes of *T. aestivum*, *Z. mays* and *H. vulgare*. The other members in Group IV were *ITPK3* genes in which *T.aestivum ITPK3* group showed more similarity with *O. sativa* and *H. vulgare*. The total six genes of *T. aestivum* are present in Group IV. This classifications of groups can also be seen in Fig 1B depicting multiple sequence alignment of protein sequences of the members of each four groups.

**Fig 1:**
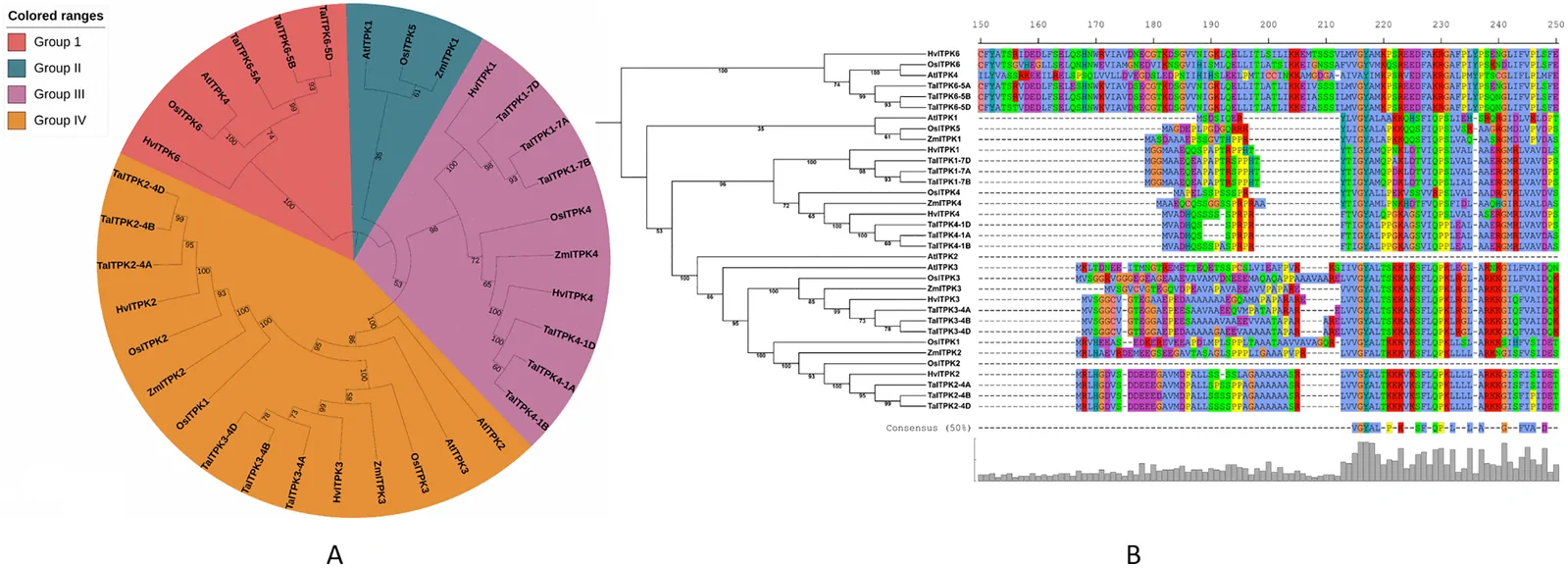
A. Comparative Phylogenetic tree of ITPK proteins of Triticum aestivum with Arabidopsis thaliana, Zea mays, Hordeum vulgare and Oryza sativa. Here, the phylogenetic tree was divided into four groups based on different ITPKs genes. Each color showed different group. The numbers adjacent to the branches represent bootstrap values ranges from 35 to 100%. **B**. Multiple sequence alignment of ITPK protein sequences of different species included in each four phylogenetic groups.

### 3.2. Gene structure, conserved motif & domain analysis of *TaITPK* gene family

To study the structural conservation and potential functional divergence among the *ITPK* gene family members, comprehensive motifs, domains and gene structure analysis were performed among *ITPK* gene family members of *T. aestivum*, *A. thaliana*, *O. sativa*, *H. vulgare* and *Z. mays*. The results strongly support the phylogenetic classification and provide evidence of evolutionary conservation of gene family and subgroups division.

A total 10 conserved motifs have been identified among which many motifs like motif 1, 2, 3, and 7 present throughout in all member of *ITPK* family (Fig 2A). These conserved regions might indicate their role in inositol phosphate kinase activity, including ATP-binding and substrate recognition sites. The *ITPK2* and *ITPK3* showed a highly conserved motif pattern arranged in same order and position showing highly conserved structural and functional redundancy. Likewise, *ITPK1* and *ITPK4* gene family member showed conserved pattern typically arranged in same order and depicting their evolutionary conservation [42]. Furthermore, members of *ITPK6* group showed contrast with others with different arrangements and losses of motifs suggesting functional divergence or adaptation.

**Fig 2:**
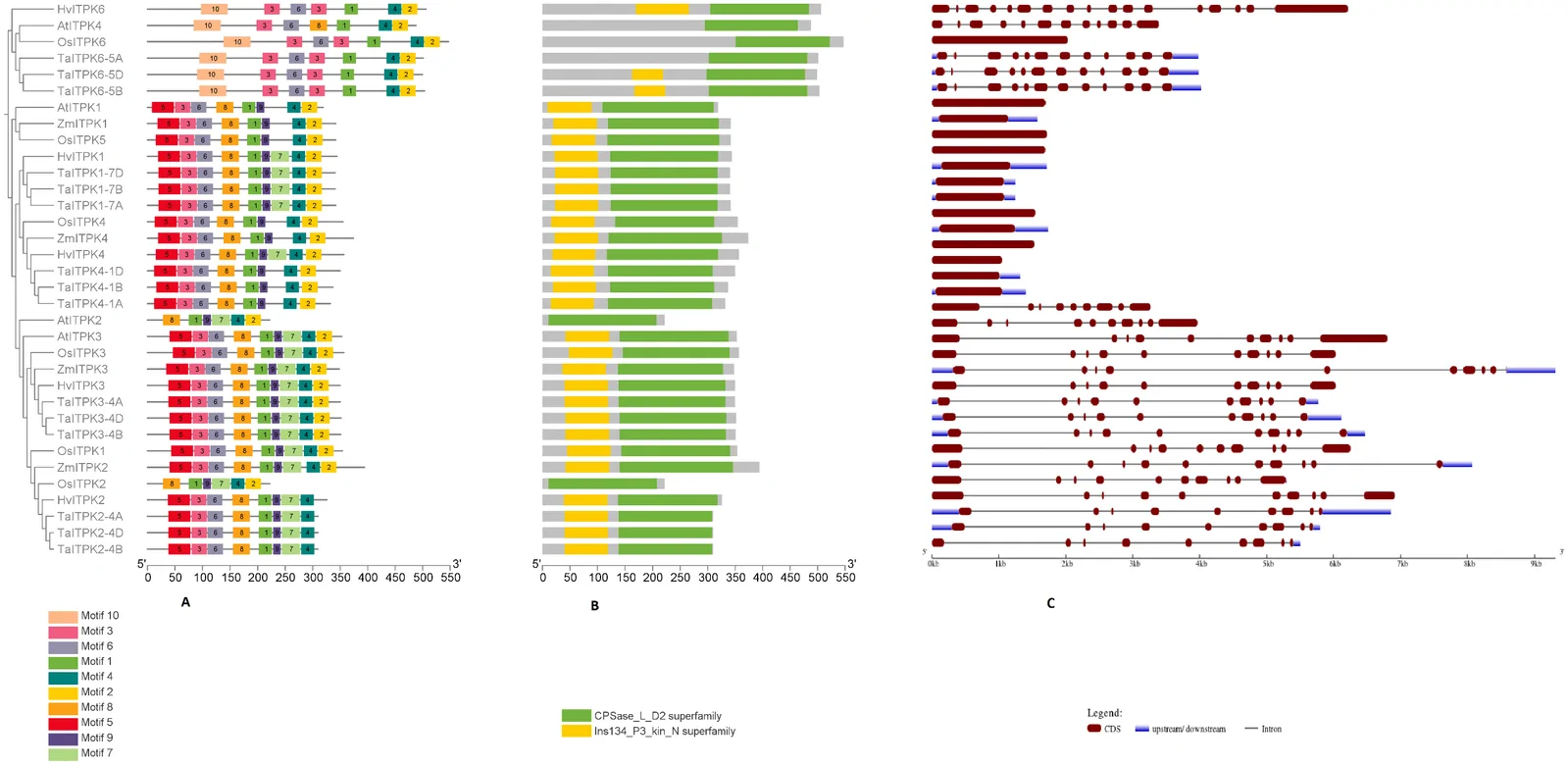
A. Conserved motifs in TaITPK protein family B. Conserved Domains in TaITPK protein family members. **C**. Gene structure of TaITPK gene family.

Functional domain analysis (Fig 2B) which revealed multiple conserved domains in *ITPK* gene family. Two super domain families present were Ins 134_P3_kin_N superfamily (inositol 1, 3, 4-trisphosphate 5/6-kinase proteins) an ATP grasp domain that plays a crucial intermediary role by catalyzing the conversion of inositol trisphosphate (IP_3_) to inositol pentaphosphate (IP_5_) via sequential phosphorylation [12] and CPase_L_D2 (Carbamoyl-phosphate synthase L chain) an ATP binding domain which catalyzes the ATP-dependent synthesis of carbamyl-phosphate from glutamine or ammonia and bicarbonate [43].

The diversity in exon–intron structures within a gene family provides valuable insights into the evolutionary history and functional roles of its member [44]. To examine the structural features of *ITPK* genes, exon-intron were analyzed and the results (Fig 2 C) revealed that the four group of *ITPK* genes showed variation among them but similarity within the same group except few of the genes. The homeologous genes in wheat showed highly conserved structure within each group and had nearly similar exon-intron arrangement which indicates their conserved function among sub genomes and originated from same ancestral gene prior to polyploidization [45]. Furthermore, in case of *ITPK1* group, only one exon presents in all species including monocots and dicots but varying in length of the exon. The *TaITPK1* showed similar length of exon of size 1kb with *Zea mays* but *AtITPK1*, O*sITPK1*, *HvITPK1* have longer exon size >1kb. Interestingly, *OsITPK5* showed similarity with *AtITPK1* and *HvITPK1* and motif pattern depicts the similar kind of interpretation. The *OsITPK1* gene showed more number of exons and introns not similar to other species indicating moderate complexity. Moreover, *TaITPK4* genes and *ZmITPK1* showed similarity with *ITPK1* group of wheat having single exon and size of 1kb. The *HvITPK4* also have single exon structure but size vary compared to other species. The *OsITPK4* gene also have only one exon similar to *HvITPK4* with same size. The slight variation in exon length indicated species specific coding sequences or variation in untranslated region (UTR) that would impact mRNA stability or translation efficiency sequence adaptions [46].

### 3.3. Comparative synteny analysis of *ITPK* gene family across different species

To study the evolutionary relationship among *ITPK* gene family members, we constructed an interspecies synteny analysis of 5 species comparing *Triticum aestivum* with other three monocotyledons species, i.e., *Hordeum vulgare*, *Oryza sativa*, *Zea mays* and one dicotyledon, i.e., *Arabidopsis thaliana* (Fig 3). The strength of association of *TaITPK* genes from highest to lowest with other species was: *Hordeum vulgare* [10], *Oryza sativa* [4], *Zea mays* [3] and *Arabidopsis thaliana* (0). The wheat and barley showed highest chromosome level conservation supporting known evolutionary relationships among Triticeae cereal. Furthermore, the synteny analysis of *Triticum aestivum* with *Oryza sativa* and *Zea mays* revealed limited chromosome-level collinearity may be due to extensive genome rearrangement and structural divergence following their evolutionary separation.

**Fig 3:**
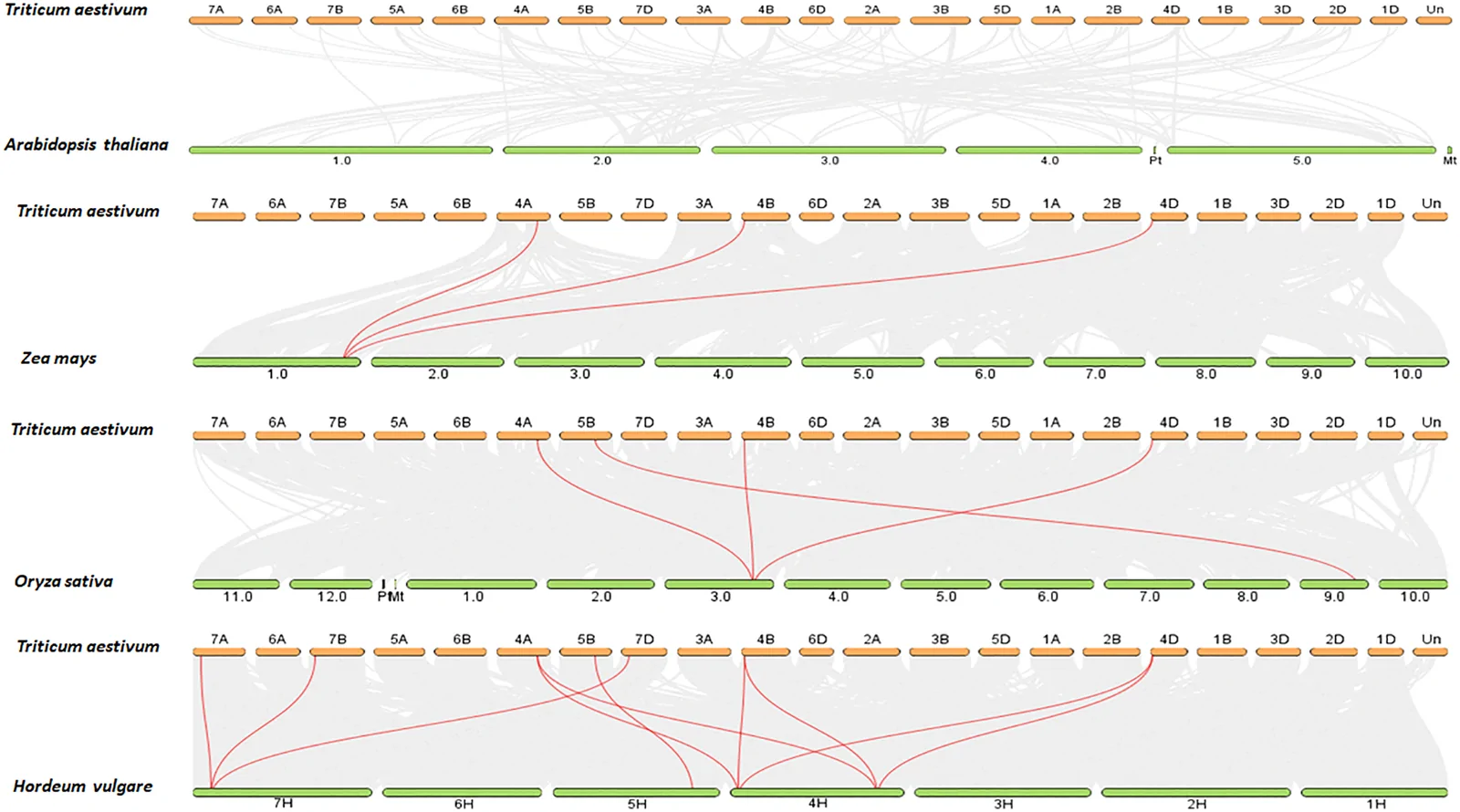
Comparative synteny analysis of *Triticum aestivum* with *A. thaliana*, *Zea mays*, *Oryza sativa* and *Hordeum vulgare.* Syntenic gene pairs are highlighted in red lines.

### 3.4. Gene localization, selection pressure and divergence analysis of *TaITPK* genes

Hexaploid wheat (*Triticum aestivum* L.) consists of three sub-genomes, i.e., A, B, and D allowing each wheat gene to have possibly three homologs, one from each set of homologous chromosomes. Genes of *TaITPK* family randomly and unevenly distributed. Out of total seven chromosomes groups in wheat, *ITPK* genes were present on chromosome 1, 4, 5 and 7 (Fig 4). The chromosome 1A, 1B, 1D carried one gene respectively. Likewise, chromosome 7A, 7B, 7D and 5A, 5B and 5D also contained one gene present each on them. The chromosome 4 contained abundance no. of genes, i.e., 6 in manner of 2 genes on each 4A, 4B and 4D respectively. None of the gene was present on chromosomes 2, 3 and 6. Thus, a total of 15 *TaITPK* genes were identified across the A, B, and D sub genomes.

**Fig 4:**
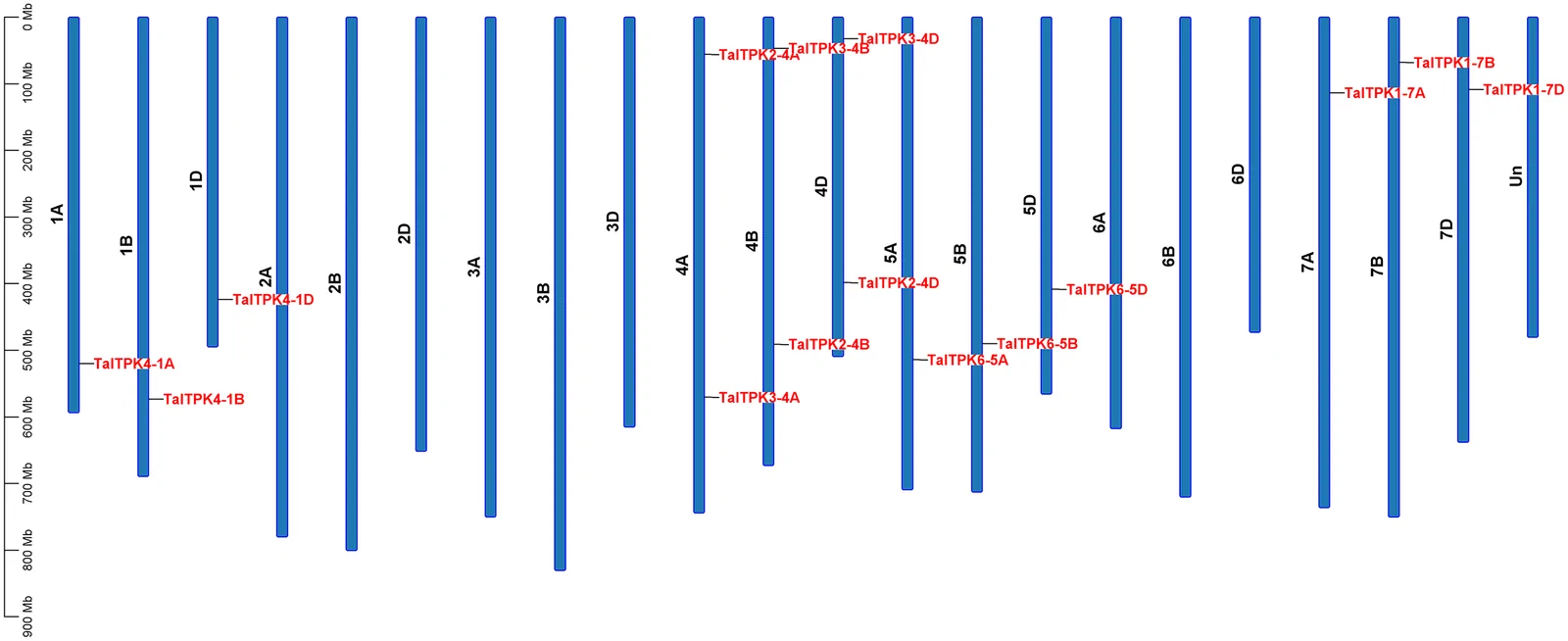
Chromosomal distribution and location of *ITPK* genes on *Triticum aestivum* genome.

Here, the identified genes have been shown in red and chromosome with grey bar numbered in red

Furthermore, to analyze the selection pressure of *ITPK* gene family in wheat, the ratio of nonsynonymous (Ka) to synonymous (Ks) substitution rates was calculated describing that how natural selection affect *ITPK* gene divergence in wheat. If the value of Ka/Ks = 1, it indicates neutral selection, where mutations are neither strongly advantageous nor disadvantageous. Ka/Ks ratio < 1 indicates purifying selection, and Ka/Ks ratio > 1 indicated positive selection [28,47]. The results revealed both positive and negative evolution in *ITPK* genes in *Triticum aestivum* (S2 Table**)** (See Supporting Information). The total 33 duplication events have been observed in *Triticum aestivum* during evolution. In addition to identifying the type of selection, the approximate divergence time between duplicated gene pairs was calculated using formula T = Ks / (2r) × 10^−6^, where T represents the divergence time in million years, Ks is the rate of synonymous substitutions per site, and r corresponds to the assumed rate of synonymous substitutions per site per year. The substitution rate of 6.5 × 10^−9^ substitutions per synonymous site per year was used as a standard reference in cereals [48]. This calculation provided insights into the evolutionary timeframe during which duplication events occurred.

### 3.5. Analysis of cis-regulating elements in the promoter of *TaITPK* family genes

The function of a gene is largely influenced by the type and abundance of cis-regulatory elements present in its promoter region [49]. To study the transcriptional regulation of *TaITPK* genes, the 1.5 kb upstream promoter regions were analyzed using PlantCARE. A wide range of cis acting elements were noted including motifs associated with hormone response, growth, development and stress response which depicted the diverse regulatory control of *TaITPK* gene family (Fig 5). The promoter region also contained TATA-box and CAAT-box which are essential elements for transcriptional machinery [50]. Moreover, an abundance of development and seed-specific elements were also observed indicating that *TaITPK* genes were also active during grain filling maturation stage that coincide to accumulation of phytic acid

**Fig 5:**
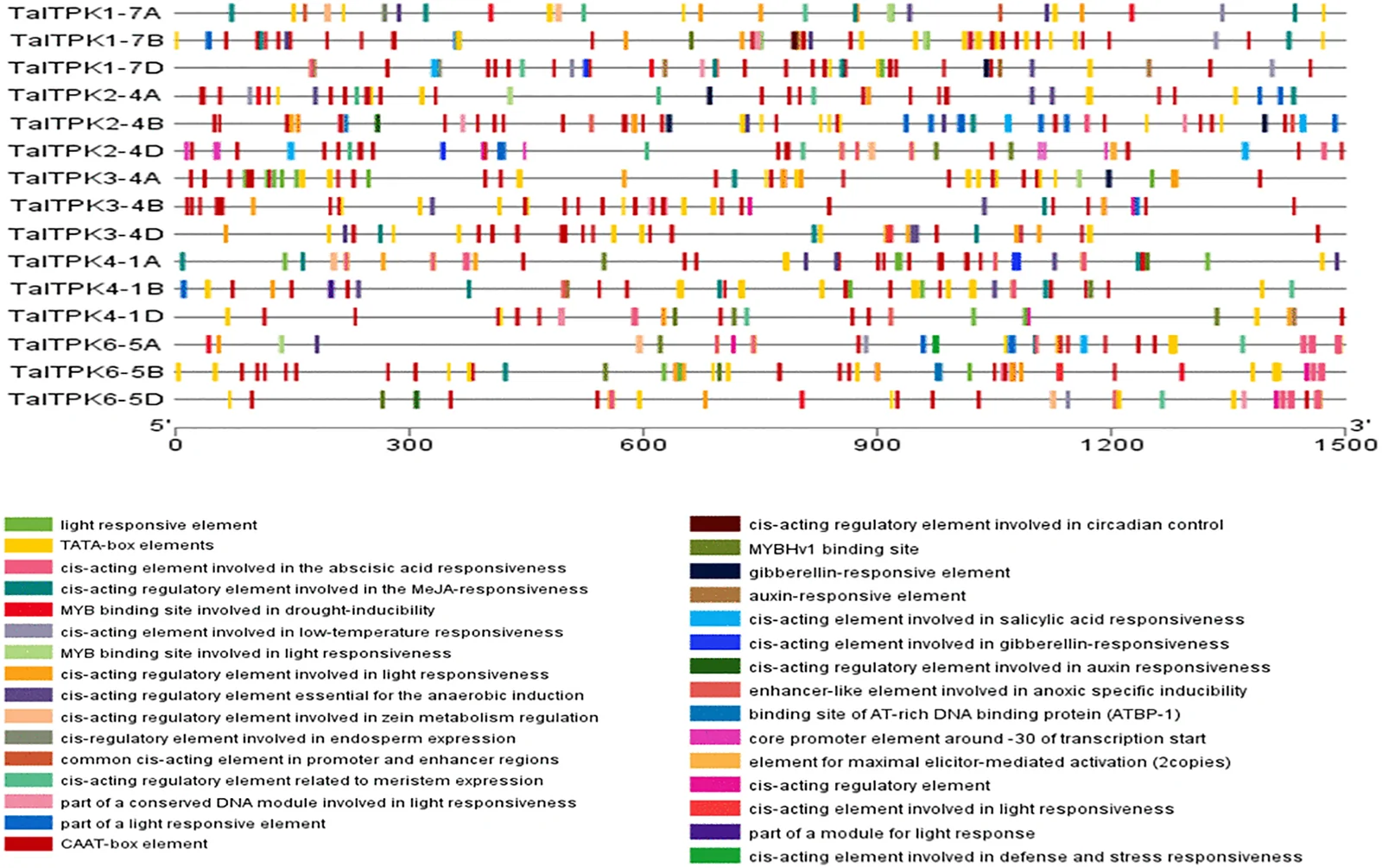
Analysis of cis-acting elements in the *TaITPK* genes promoters. The genomic sequence 1500 bp upstream of the start codon was selected as the promoter for analysis.

### 3.6. Protein modeling and protein-protein interaction

To gain insight into the structural information about inositol tris-phosphate 5/6 protein, tertiary structures of protein models were generated via Swiss model using protein sequences of 15 wheat *ITPK* genes (Fig 6) Each *ITPK* groups from 1–4,6 showed similarity within their homeologous copies present on A, B and D genome. The quality of models was evaluated through GMQE (Global Model Quality Estimate) and QMEANDisCo [32,51] where GMQE and QMEANDisCo global scores range from 0 to 1, with higher values indicating better expected model quality [52]. The results suggested strong conservation among homoeologs. As wheat has hexaploid genome, the presence of similar A, B, and D copies strongly supported the idea that these genes were retained after polyploidization and remained structurally conserved. This suggests that these homoeologs may still be functionally important and were not lost during genome evolution.

**Fig 6:**
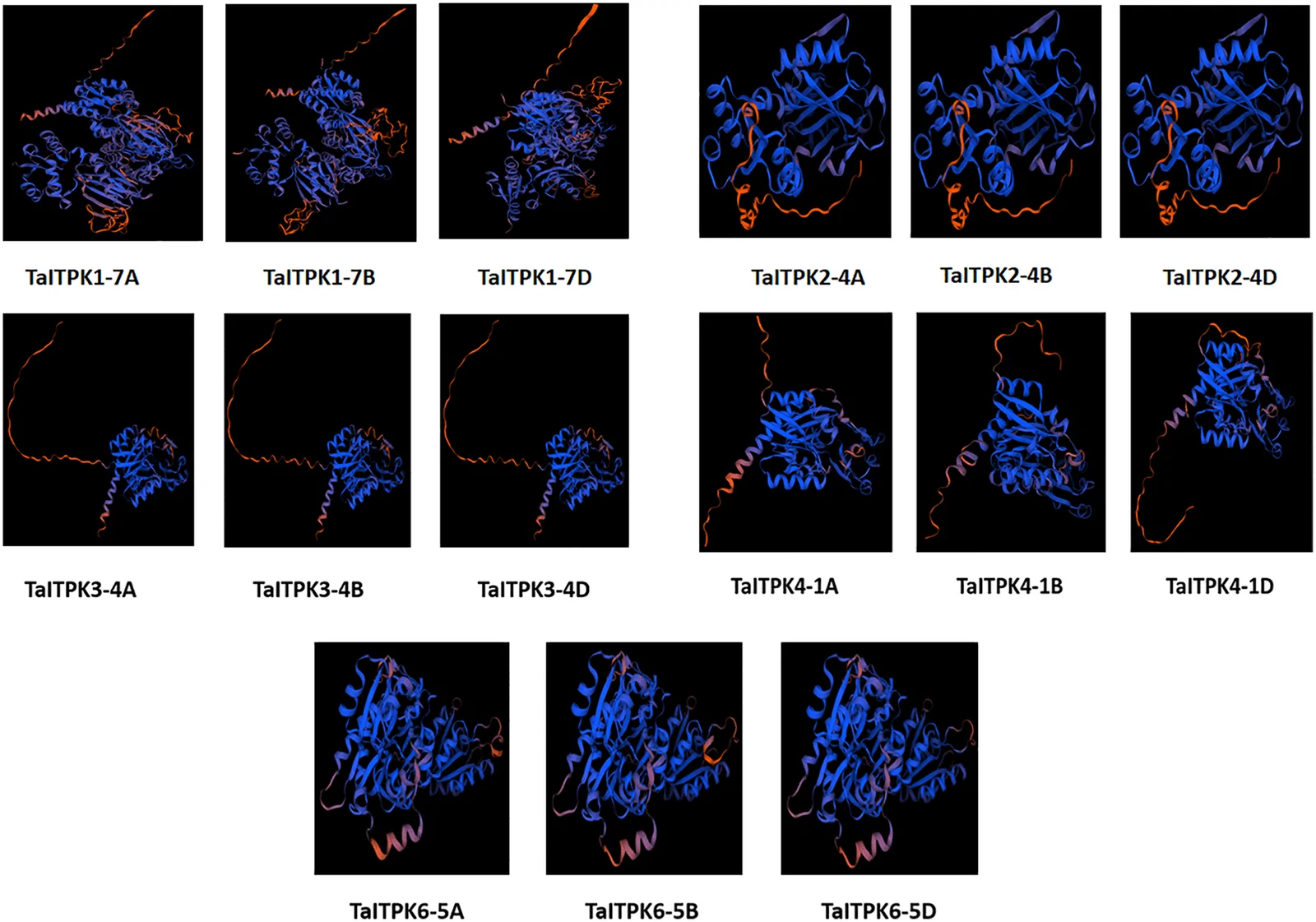
3D Protein structures of 15 *TaITPK* proteins predicted using SWISS-MODEL and the model with the best results was selected based on QMEAN and GMQE.

The interaction of wheat *ITPK* genes with other proteins was further studied (Fig 7). The Protein-Protein Interaction (PPI) network of *ITPK* protein was constructed on STRING database with highest confidence (0.900) interaction score. The results showed that all the *TaITPK* proteins showed interaction with inositol pentakisphosphate 2-kinase which phosphorylates Ins (1,3,4,5,6) P5 at position 2 to form Ins (1,2,3,4,5,6) P6. Moreover, the *TaITPK* proteins also showed protein interaction with inositol polyphosphate multikinase. The analysis showed < 1.0e-16 PPI value which indicated higher degree of statistical significance and more interaction of *TaITPK* proteins among themselves than what would be expected for a random set of proteins of the same size and degree distribution drawn from the genome. Such an enrichment indicated that the proteins were at least partially biologically connected, as a group [53].

**Fig 7:**
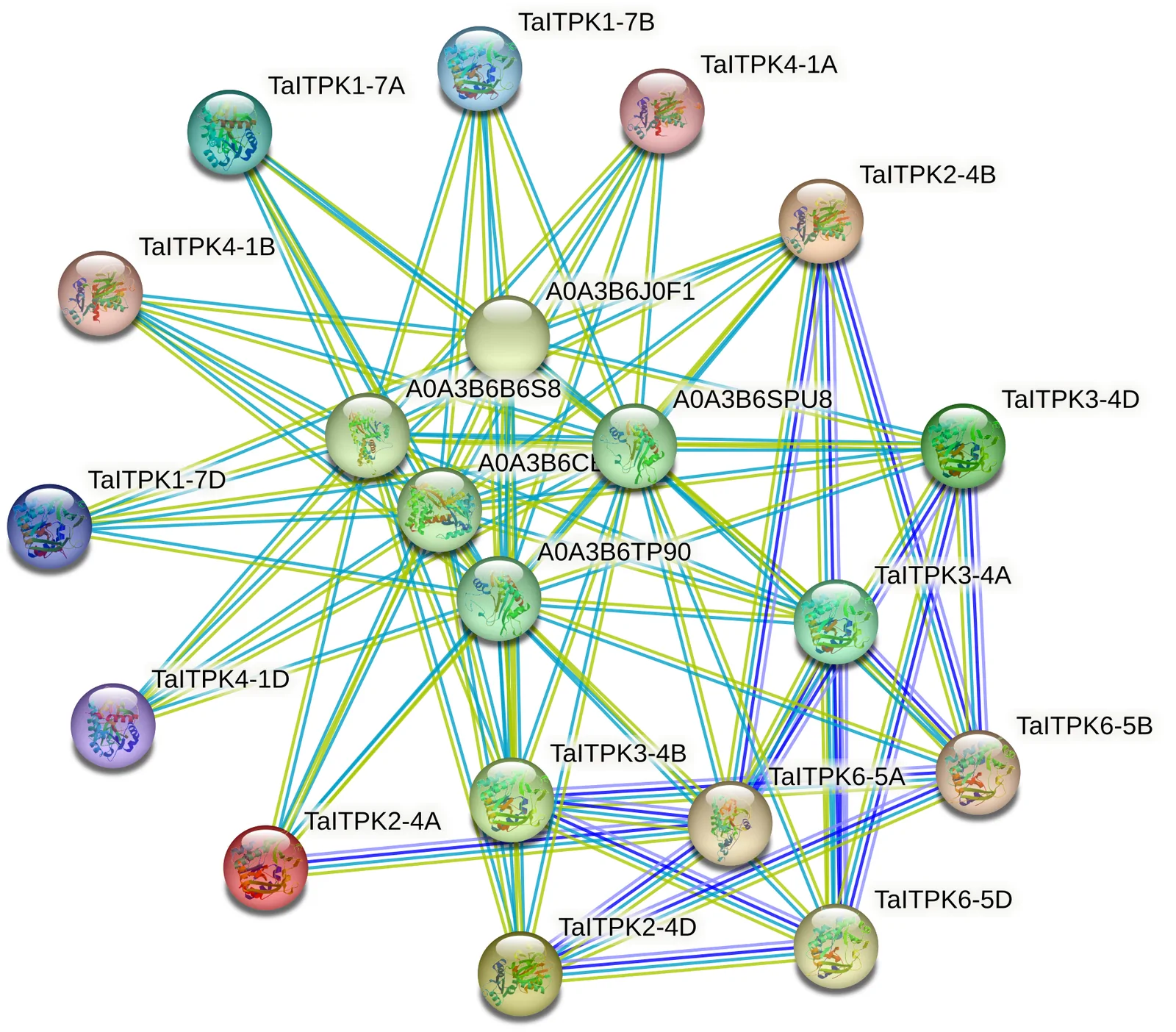
PPI network of *TaITPK* protein family with other proteins with highest confidence (0.900) interaction score. Here, *A0A3B6J0F1*, *A0A3B6B6S8*, *A0A3B6CED3* represent Inositol-pentakisphosphate 2-kinase; *A0A3B6SPU8*, *A0A3B6TP90* represent Inositol polyphosphate multikinase proteins.

### 3.7. Tissue specific in silico *TaITPK* expression analysis

The RNA sequence data of Chinese spring wheat cultivar in different plant tissues and at different growth stages were analyzed. The results (Fig 8) revealed that *TaITPK4-1A*, *TaITPK4-1B* and *TaITPK4-1D* showed highest expression as compared to other genes in stem, spike, root and grain and low to none expression was observed in leaf tissue. In case of *TaITPK3-4A*, *TaITPK3-4B* and *TaITPK3-4D* moderate expression was observed in grain_z85 stage while at growth and development stages, zero to low level of expression was observed. The expression of *TaITPK2-4A*, *TaITPK2-4B* and *TaITPK2-4D* was low except at leaf_z71 stage which showed moderate gene expression. In *TaITPK6-5A*, *TaITPK6-5B* and *TaITPK6-5D*, the expression observed was moderate at all stages except in grain filling stages. The expression of *TaITPK1-7A*, *TaITPK1-7B* and *TaITPK1-7D* showed least expression results. The results showed contradiction to Yang et al. [54] observations in which the expression of *AtITPK4* genes was lowest in root, stem, leaves and silique tissues and highest in *AtITPK3* gene. This indicated the species-specific expression pattern of genes. Stiles et al. [10] showed highest expression of *ITPK3* gene and lowest in *ITPK2* and more expression was observed in early seed development stage.

**Fig 8:**
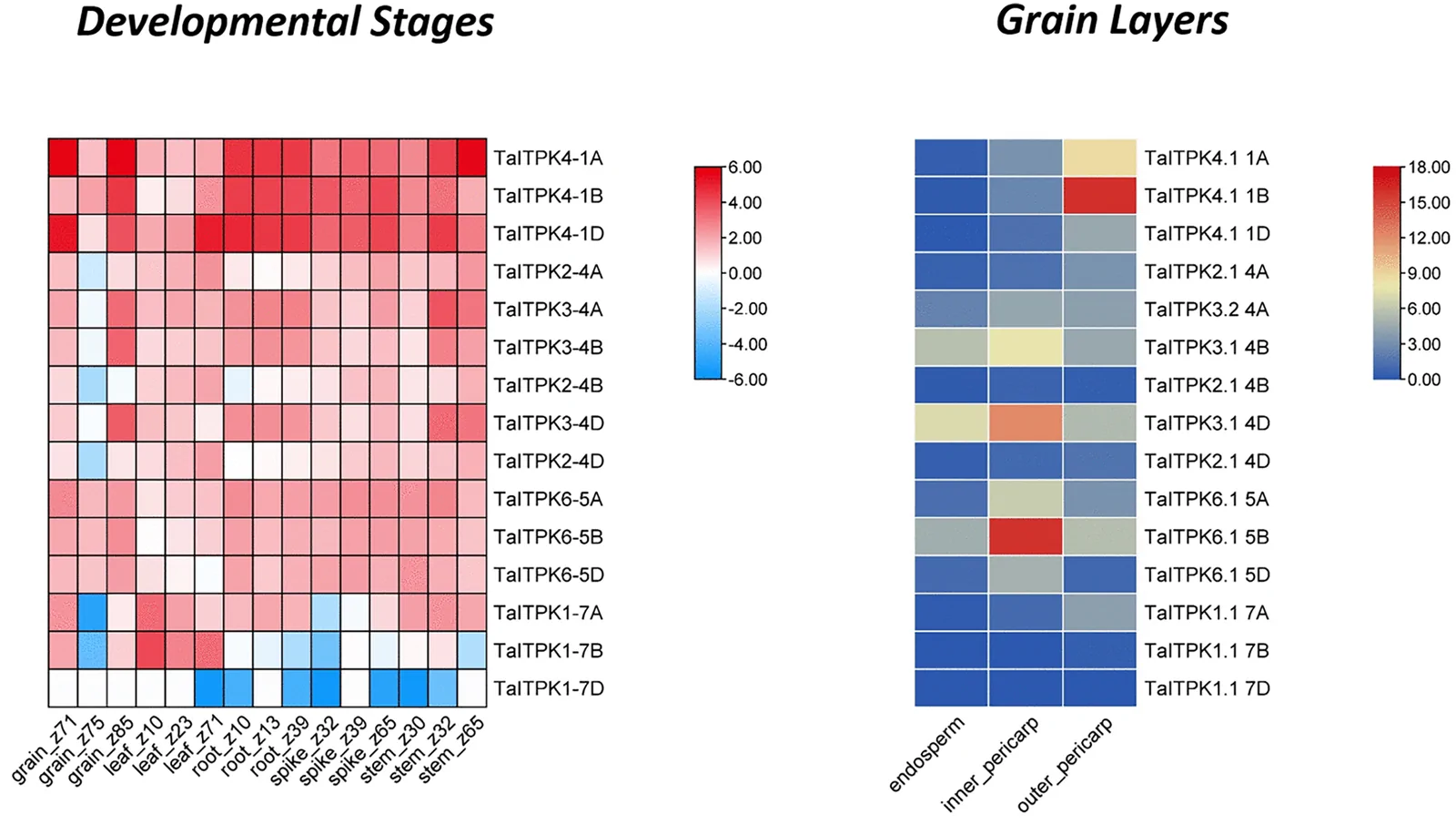
Heat map showing in silico expression analysis of *TaITPK* genes at different development stages and grain layers.

Here, Zadoks scale(Zs) stages represented as: (Seedling stage = (zS10); Three leaves = zS13; Three tillers = zS23; Spike at 1 cm = zS30; Two nodes = zS32; Meiosis = zS39; Anthesis = zS65; 2 DAA = zS71; 14 DAA = zS75; 30 DAA = zS85 10). The bar scale at left showed the levels of expression: red color for highest expression level and blue color showed lowest expression level.

### 3.8. Expression patterns of *TaITPK* genes in response to FeSO_4_ and ZnSO_4_

To study the response of Inositol tetrkisphosphate (ITPK) genes involved in biosynthetic pathway of phytic acid, the relative expression analysis of four ITPK genes (ITPK2-4A, ITPK3-4D, ITPK4-1A, and ITPK6-5A) (as shown in Fig 9) were selected on the basis of in silico expression profiling. As similar in silico expressions were observed among the A, B, and D subgenome homoeologs, one representative member from each homoeologous group was chosen for preliminary expression analysis. ITPK1 was not included due to its comparatively weak overall in silico expression. However, this does not exclude the possibility that *ITPK1* may show higher expression at other developmental stages, in specific tissues, or under conditions not examined in the present study. Furthermore, the selected genes were analyzed in two wheat genotypes, Fateh-Jang and Akbar-19 under control and minerals treated conditions at grain filling stage. The results revealed treatment-dependent expression pattern among the selected ITPK genes in both wheat genotypes at the grain filling stage. *TaITPK3-4D* showed comparatively higher relative expression under FeSO_4_, ZnSO_4_, and combined FeSO_4_ + ZnSO_4_ treatments in both Fateh-Jang and Akbar-19. *TaITPK2-4A* showed relatively low expression under individual treatments but showed higher relative expression under the combined treatment, especially in Akbar-19 genotype. In contrast, *TaITPK4-1A* showed relatively higher expression under FeSO_4_, especially in Fateh-Jang, whereas *TaITPK6-5A* showed increased in relative expression under combined treatment in Fateh-Jang only. Similar lower expression pattern of *ITPK6* gene was observed in Arabidopsis *ITPK4* in silique development stage by Yang et al*.* [54].

**Fig 9:**
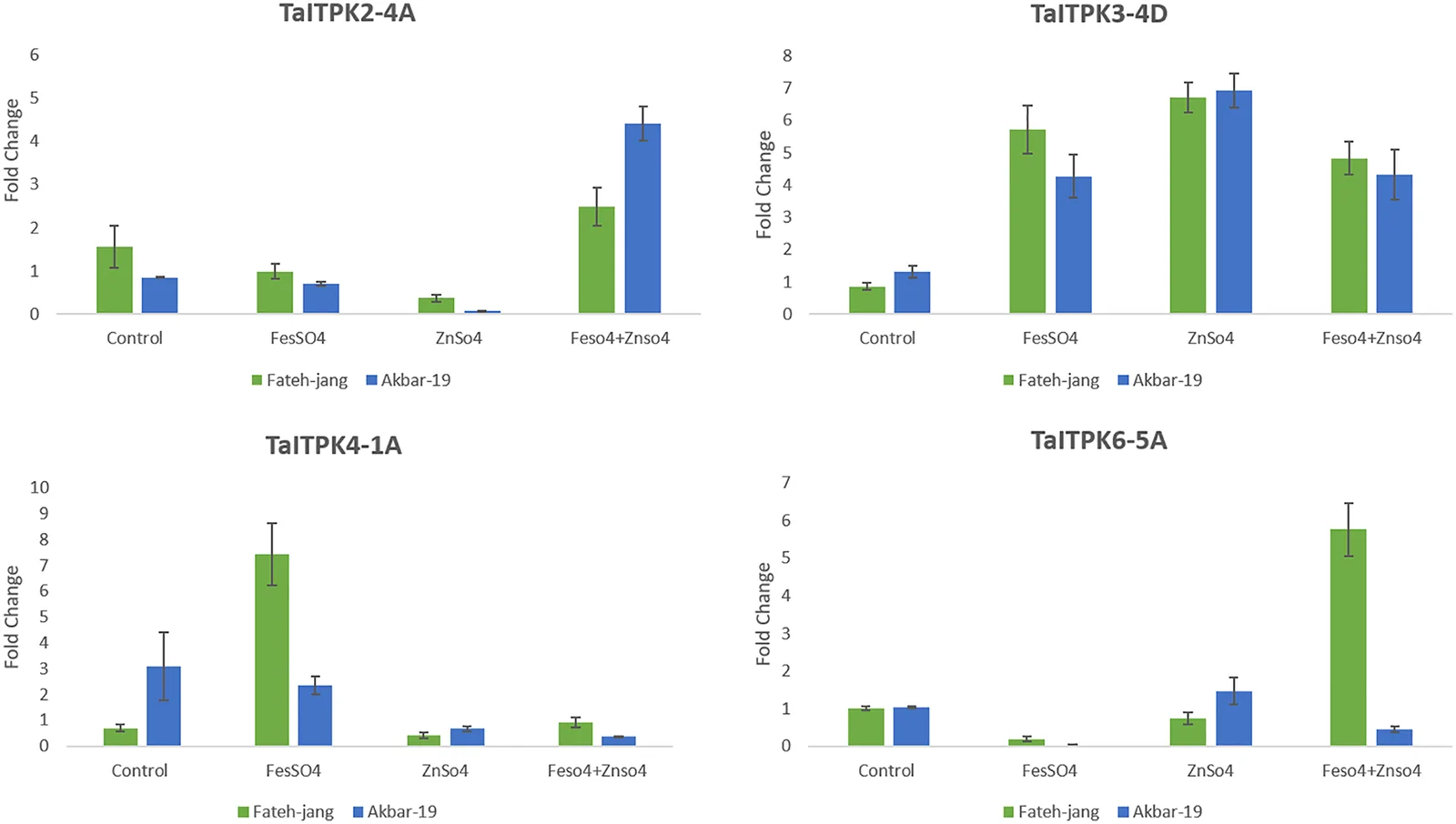
Preliminary descriptive relative expression levels of *TaITPK2-4A*, *TaITPK3-4D*, *TaITPK4-1A* and *TaITPK6-5A* genes under control and mineral treatment. qRT-PCR was used to examine the expression levels of spikes at grain filling stage in triplicate technical repeats. Bars represent mean relative expression ± SE of three technical measurements from one biological sample per genotype × treatment combination.

## 4. Discussion

The genome wide analysis identified *15* Inositol tetrakisphosphate (*ITPK*) genes which were distributed across the A, B and D sub-genomes of hexaploid wheat (*Triticum aestivum* L.) The presence of several homoelogous copies within each clade showed extensive gene expansion which were the results of polyploidization and tandem duplication [55–57]. The conservation of exon-intron arrangements and motifs among homoeologs depicted functional conservation during evolution while, some small differences among the gene copies indicate that they might have developed slightly different roles, helping the plant adapt to different growth or stress conditions. All *TaITPK* proteins contained the inositol [1,3,4] P3 5/6-kinases (*ITPKs*) domain, which is a conserved domain responsible for adding phosphate groups to inositol phosphate molecules to convert inositol triphosphate (InsP3) to inositol pentaphosphate (InsP5) via sequential phosphorylation [58]. This reaction is a key step in the phytate (inositol hexakisphosphate, IP₆) biosynthesis pathway. The conservation of this domain confirms that all identified wheat *ITPK* genes have similar catalytic functions to those found in rice and Arabidopsis, which are also involved in phosphate metabolism.

Furthermore, the mapping of *TaITPK* genes on wheat chromosomes showed that they were unevenly distributed across different chromosomes, but their positions correspond well to those found in other cereal crops like barley, rice and maize. This showed that these genes have evolutionarily conserved across cereals. The phylogenetic tree grouped *TaITPK* genes into several clades with their homologs from rice, maize, and barley, indicating that the *ITPK* gene family existed before the divergence of these species [47] and then expanded in wheat through polyploidization. Comparative synteny mapping between wheat and other cereal species revealed strong collinearity with rice, maize, and barley, confirming a conserved evolutionary path among monocots. These conserved genomic regions highlighted the functional stability of *ITPK* genes, suggesting that their enzymatic role in the inositol phosphate pathway has remained under strong purifying selection pressure throughout cereal evolution. In contrast, limited collinearity was observed between *Triticum aestivum* and the dicot *Arabidopsis thaliana*, which is consistent with the deep evolutionary divergence between monocots and dicots. The absence of syntenic relationships with Arabidopsis highlighted that *ITPK* gene family expansion and diversification were shaped primarily within monocot-specific evolutionary pathways.

The cis-regulatory (promoter) analysis revealed the presence of a numerous cis-regulatory elements that were responsible for controlling *TaITPK* gene expression during different developmental stages and stress conditions. Importantly, several growth and development related cis-elements such as the GCN4_motif, O2-site, and CAAT-box were detected. The GCN4_motif is an endosperm-specific element that activates genes during seed development and storage compound formation [59]. It was mostly found in promoter region of *TaITPK1* genes that indicated the transcriptionally activation of this gene group during grain development. The O2-site were linked with seed maturation and protein synthesis [60,61] and the CAAT-box was generally involved in meristem and tissue differentiation [62,63] which may contribute to expression regulation during early development stage and reproductive tissue formation. In addition to developmental elements, several hormonal and stress-responsive elements, including ABRE (abscisic acid response), TGACG-motif (MeJA response), and TCA-element (salicylic acid response), were also detected. These cis-elements indicated the potential roles of *TaITPK* genes in phosphate signaling and abiotic stress regulation, as hormonal crosstalk often influences inositol phosphate metabolism under environmental challenges [64].

The in-silico expression analysis revealed that several *TaITPK* genes were highly expressed in spike and grain tissues, especially during the reproductive and grain-filling stages [53]. This expression pattern coincided with the grain developmental period during which phytic acid accumulation is known to occur, suggesting a possible role of selected *TaITPK* genes in phytic acid metabolism during seed development [65]. Furthermore, preliminary qRT-PCR profiling indicated treatment dependent differences in the relative expression patterns of selected *TaITPK* genes under Fe and Zn supplementation. Among the genes examined, *TaITPK3-4D* showed comparatively higher relative expression under both Fe and Zn treatments, whereas *TaITPK6-5A* displayed a distinct expression pattern under the combined Fe and Zn treatment. These observations suggest that individual *TaITPK* members may respond differently to mineral availability. However, because the qRT-PCR measurements were based on technical replicates without independent biological replication, these expression patterns should be interpreted descriptively and require confirmation in biologically replicated experiments before any functional or metabolic conclusions can be drawn.

Phytate (inositol hexakisphosphate, IP₆) is the primary phosphorus storage compound in cereal grains [66], but it also functions as a strong chelator of divalent metal ions such as iron (Fe²⁺) and zinc (Zn²⁺), thereby reducing their bioavailability in humans and animals which mainly contributed towards micronutrient deficiencies in consumers [15]. The combined presence of seed-specific cis-elements and high expression of *TaITPK* genes in grains suggested that these genes play a critical role in phytate accumulation and consequently influence nutritional quality. Targeted manipulation of *TaITPK* genes offers a practical molecular strategy to address this problem. The identification of endosperm-active *TaITPK* or seed specific gene homologs provides suitable targets for CRISPR/Cas9-mediated gene knockout or silencing, allowing the development of low-phytate wheat lines without affecting plant growth or yield. Previous studies in rice and maize also showed that editing or silencing of *ITPK* genes significantly reduced seed phytate levels and improved mineral bioavailability [67–69]. Understanding this division of function will help breeders/ biotechnologists to target specific *TaITPK* genes without disturbing essential phosphate signaling pathways in other tissues. Although *ITPK* genes are functionally associated with the phytic acid biosynthetic pathway, phytic acid content was not directly measured in the present study. Therefore, the observed *TaITPK* expression patterns cannot be interpreted as direct evidence of changes in phytic acid accumulation. The possible relationship between *TaITPK* expression and phytic acid accumulation remains a hypothesis based on previous studies and requires validation through direct, biologically replicated phytic acid measurements.

## 5. Conclusion

In this study, we conducted a comprehensive genome-wide analysis of the *TaITPK* gene family, examining conserved domains, gene structure, protein motifs, chromosomal locations, selection pressure analysis, protein-protein interaction and expression profiles in different tissues at different growth stages which play an important role in synthesis of phytic acid. This in-depth study provides a foundation for researchers to better understand these genes and identify promising candidate genes for developing low-phytate crops by using advanced biotechnological tools, such as genome editing. Consequently, it paves the way for enhancing the nutritional quality of common wheat (*T. aestivum*) and other staple food crops.

## Supporting information

S1 Table Characteristics of *TaITPK* proteins identified in *Triticum aestivum.* (DOCX)

S2 Table Ka/Ks ratios and divergence time of paralogous ITPK gene pairs. (DOCX)

S3 Table Motif sequence and their width. (DOCX)

S4 Table Gene Ids of ITPK proteins of *O. sativa*, *H. vulgare* and *Z. mays.* (DOCX)

S5 Table List of primers used in qRT-PCR analysis. (DOCX)

S1 File Cis-regulatory elements identified in the promoter regions of TaITPK genes. (XLSX)

S2 File Relative expression calculations for selected TaITPK genes. (XLSX)
