# Supplementary material for: Genome wide identification and analysis of inositol tetrakisphosphate kinase (ITPK) gene family in Triticum aestivum L
Source: PLoS One. 2026 Sep 29;21(9):e0359528. doi: 10.1371/journal.pone.0359528 (PMC13623094; doi:10.1371/journal.pone.0359528)
Supplement: Supplementary file 1 [file pone.0359528.s001.docx]

**S Table 1.** Characteristics of *TaITPK* proteins identified in *Triticum aestivum*

| **Gene ID** | **Rename ID** | **Chr ID** | **Start** | **End** | **Strand** | **AA** | **Weight(kds)** | **pI** | **GRAVY** | **Subcellular location** |
| --- | --- | --- | --- | --- | --- | --- | --- | --- | --- | --- |
| TraesCS1A02G330900 | TaITPK4-1A | 1A | 5.2E+08 | 5.2E+08 | + | 332 | 35.50067 | 5.07 | 0.131 | Cytoplasm |
| TraesCS1B02G344900 | TaITPK4-1B | 1B | 5.73E+08 | 5.73E+08 | - | 337 | 35.82899 | 4.98 | 0.156 | Cytoplasm,  Chloroplast |
| TraesCS1D02G333500 | TaITPK4-1D | 1D | 4.24E+08 | 4.24E+08 | + | 350 | 37.1135 | 5 | 0.148 | Plamsma membrane,  Cytoplasm |
| TraesCS4A02G059500 | TaITPK2-4A | 4A | 55912521 | 55919258 | + | 310 | 34.20648 | 5.52 | -0.077 | Cytoplasm |
| TraesCS4A02G257700 | TaITPK3-4A | 4A | 5.7E+08 | 5.7E+08 | - | 214 | 23.88068 | 7.86 | -0.037 | Mitochondria,  Extracellular |
| TraesCS4B02G056800 | TaITPK3-4B | 4B | 46615028 | 46621497 | + | 351 | 38.6105 | 6.77 | -0.145 | Cytoplasm |
| TraesCS4B02G235100 | TaITPK2-4B | 4B | 4.91E+08 | 4.91E+08 | + | 310 | 34.26954 | 5.45 | -0.09 | Cytoplasm |
| TraesCS4D02G057100 | TaITPK3-4D | 4D | 32229706 | 32238262 | + | 352 | 38.65845 | 6.77 | -0.171 | Cytoplasm |
| TraesCS4D02G236500 | TaITPK2-4D | 4D | 3.98E+08 | 3.98E+08 | + | 310 | 34.22553 | 5.55 | -0.081 | Cytoplasm |
| TraesCS5A02G305400 | TaITPK6-5A | 5A | 5.14E+08 | 5.14E+08 | - | 501 | 55.3479 | 5.15 | 0.053 | Cytoplasm |
| TraesCS5B02G305900 | TaITPK6-5B | 5B | 4.9E+08 | 4.9E+08 | - | 503 | 55.49705 | 5.23 | 0.022 | Cytoplasm |
| TraesCS5D02G312400 | TaITPK6-5D | 5D | 4.08E+08 | 4.08E+08 | - | 499 | 54.98837 | 5.13 | 0.03 | Cytoplasm |
| TraesCS7A02G158400 | TaITPK1-7A | 7A | 1.13E+08 | 1.13E+08 | + | 342 | 36.72189 | 4.95 | 0.03 | Chloroplast,  Cytoplasm,  Mitochondra |
| TraesCS7B02G063100.1 | TaITPK1-7B | 7B | 67818474 | 67819722 | + | 341 | 36.62368 | 4.83 | 0.012 | Cytoplasm |
| TraesCS7D02G159400.1 | TaITPK1-7D | 7D | 1.09E+08 | 1.09E+08 | + | 341 | 36.67378 | 4.84 | 0.032 | Chloroplast,  Cytoplasm,  Mitochondra |
