# Supplementary material for: Genome wide identification and analysis of inositol tetrakisphosphate kinase (ITPK) gene family in Triticum aestivum L
Source: PLoS One. 2026 Sep 29;21(9):e0359528. doi: 10.1371/journal.pone.0359528 (PMC13623094; doi:10.1371/journal.pone.0359528)
Supplement: Supplementary file 2 [file pone.0359528.s002.docx]

**S Table 2.**  Ka/Ks ratios and divergence time of paralogous ITPK gene pairs

| **Paralogue 1** | **Paralogue 2** | **ks** | **ka** | **ka/ks** | **T(MYA)** |
| --- | --- | --- | --- | --- | --- |
| TaITPK4-1A | TaITPK4-1B | 0.097 | 0.006 | 0.062249135 | 3.18×10^-6^ |
| TaITPK4-1A | TaITPK4-1D | 0.019 | 0.017 | 0.92203498 | 6.20×10^-17^ |
| TaITPK4-1A | TaITPK1-7A | 0.343 | 0.260 | 0.756678236 | 1.13×10^-15^ |
| TaITPK4-1A | TaITPK1-7B | 0.354 | 0.258 | 0.728802542 | 1.16×10^-15^ |
| TaITPK4-1A | TaITPK1-7D | 0.370 | 0.248 | 0.669625838 | 1.22×10^-15^ |
| TaITPK4-1B | TaITPK4-1D | 0.096 | 0.016 | 0.171091209 | 3.17×10^-16^ |
| TaITPK4-1B | TaITPK1-7A | 0.343 | 0.267 | 0.776553673 | 1.13×10^-15^ |
| TaITPK4-1B | TaITPK1-7B | 0.343 | 0.264 | 0.768727909 | 1.13×10^-15^ |
| TaITPK4-1B | TaITPK1-7D | 0.375 | 0.254 | 0.677694359 | 1.23×10^-15^ |
| TaITPK4-1D | TaITPK1-7A | 0.334 | 0.265 | 0.792953244 | 1.10×10^-15^ |
| TaITPK4-1D | TaITPK1-7B | 0.338 | 0.266 | 0.787209403 | 1.11×10^-15^ |
| TaITPK4-1D | TaITPK1-7D | 0.357 | 0.259 | 0.725745979 | 1.17×10^-15^ |
| TaITPK2-4A | TaITPK3-4A | 1.847 | 0.211 | 0.114417618 | 6.08×10^-15^ |
| TaITPK2-4A | TaITPK3-4B | 1.699 | 0.212 | 0.125011432 | 5.59×10^-15^ |
| TaITPK2-4A | TaITPK2-4B | 0.015 | 0.005 | 0.333323307 | 4.98×10^-17^ |
| TaITPK2-4A | TaITPK3-4D | 1.688 | 0.216 | 0.127817714 | 5.56×10^-15^ |
| TaITPK2-4A | TaITPK2-4D | 0.020 | 0.003 | 0.166097616 | 6.65×10^-17^ |
| TaITPK3-4A | TaITPK3-4B | 0.060 | 0.000 | 0 | 1.99×10^-16^ |
| TaITPK3-4A | TaITPK2-4B | 1.853 | 0.213 | 0.115175287 | 6.10×10^-15^ |
| TaITPK3-4A | TaITPK3-4D | 0.030 | 0.003 | 0.114043901 | 9.78×10^-17^ |
| TaITPK3-4A | TaITPK2-4D | 1.847 | 0.211 | 0.114417618 | 6.08×10^-15^ |
| TaITPK3-4B | TaITPK2-4B | 1.704 | 0.215 | 0.125894592 | 5.61×10^-15^ |
| TaITPK3-4B | TaITPK3-4D | 0.055 | 0.003 | 0.061468968 | 1.81×10^-16^ |
| TaITPK3-4B | TaITPK2-4D | 1.699 | 0.212 | 0.125011432 | 5.59×10^-15^ |
| TaITPK2-4B | TaITPK3-4D | 1.694 | 0.218 | 0.128706176 | 5.58×10^-15^ |
| TaITPK2-4B | TaITPK2-4D | 0.005 | 0.002 | 0.334821572 | 1.64×10^-17^ |
| TaITPK3-4D | TaITPK2-4D | 1.688 | 0.216 | 0.127817714 | 5.56×10^-15^ |
| TaITPK6-5A | TaITPK6-5B | 0.052 | 0.013 | 0.250958769 | 1.71×10^-16^ |
| TaITPK6-5A | TaITPK6-5D | 0.082 | 0.010 | 0.118562101 | 2.71×10^-16^ |
| TaITPK6-5B | TaITPK6-5D | 0.064 | 0.003 | 0.05072135 | 2.10×10^-16^ |
| TaITPK1-7A | TaITPK1-7B | 0.094 | 0.009 | 0.091328367 | 3.10×10^-16^ |
| TaITPK1-7A | TaITPK1-7D | 0.094 | 0.012 | 0.128155518 | 3.10×10^-16^ |
| TaITPK1-7B | TaITPK1-7D | 0.076 | 0.011 | 0.148687999 | 2.49×10^-16^ |
