# Supplementary material for: Genome wide identification and analysis of inositol tetrakisphosphate kinase (ITPK) gene family in Triticum aestivum L
Source: PLoS One. 2026 Sep 29;21(9):e0359528. doi: 10.1371/journal.pone.0359528 (PMC13623094; doi:10.1371/journal.pone.0359528)
Supplement: Supplementary file 3 [file pone.0359528.s003.docx]

**S Table3. Motif sequence and their width**

| **Motif No.** | **Sequence** | **Width** |
| --- | --- | --- |
| Motif 1 | PPLVLQEFVNHGGVLFKVYVVGDAI | 25 |
| Motif 2 | TRDRYYIIDINYFPGYGKMPGYEHMFTDW | 29 |
| Motif 3 | DQGPFDIIIHKLTGKEWQRQLEEYHETHP | 29 |
| Motif 4 | VEEVARELRRRLGLHLFNFDMIREH | 25 |
| Motif 5 | RYTIGYAMTPKKAKSFIQPKLRALARKKGMRFVAIDQSRPL | 41 |
| Motif 6 | EVCVVDPPNAIEHLLNRQSMLQEVSELNC | 29 |
| Motif 7 | NNEGIYRFPRVSCAAATADHADLDPHIAELPPRP | 34 |
| Motif 8 | DPSSIPTAVMMAGLTFPLIAKPLWVDGTAKSH | 32 |
| Motif 9 | CVRRFSLPDVNTYDL | 15 |
| Motif 10 | MLEWSRTSSCFYATSRVDEDLFSELQSHNWKVIAVDNECGTKDSGVVNIG | 50 |
