# Supplementary material for: Genome wide identification and analysis of inositol tetrakisphosphate kinase (ITPK) gene family in Triticum aestivum L
Source: PLoS One. 2026 Sep 29;21(9):e0359528. doi: 10.1371/journal.pone.0359528 (PMC13623094; doi:10.1371/journal.pone.0359528)
Supplement: Supplementary file 4 [file pone.0359528.s004.docx]

**S Table 4 Gene Ids of ITPK proteins of *O. sativa*, *H. vulgare* and *Z. mays***

| **GENE ID** | **Rename ID** |
| --- | --- |
| LOC4347947 | OsITPK1 |
| LOC4332146 | OsITPK2 |
| 4333967 | OsITPK3 |
| 4329322 | OsITPK4 |
| 4349492 | OsITPK5 |
| 4347597 | OsITPK6 |
| 123436322 | HvITPK1 |
| 23449242 | HvITPK2 |
| 123447636 | HvITPK3 |
| 123410001 | HvITPK4 |
| 123399966 | HvITPK6 |
| Zm00001eb024800_T001 | ZmITPK1 |
| Zm00001eb399350_T001 | ZmITPK2 |
| Zm00001eb055860_T001 | ZmITPK3 |
| Zm00001eb240900_T001 | ZmITPK4 |
| 831539 | AtITPK1 |
| 829519 | AtITPK2 |
| 826367 | AtITPK3 |
