# Supplementary material for: Genome wide identification and analysis of inositol tetrakisphosphate kinase (ITPK) gene family in Triticum aestivum L
Source: PLoS One. 2026 Sep 29;21(9):e0359528. doi: 10.1371/journal.pone.0359528 (PMC13623094; doi:10.1371/journal.pone.0359528)
Supplement: Supplementary file 5 [file pone.0359528.s005.docx]

**S Table 5. List of primers used in qRT-PCR analysis.**

| **Gene ID** | **Rename ID** | **Primer** | **Sequence (5’-3’)** |
| --- | --- | --- | --- |
| TraesCS5A02G305400 | TaITPK6-5A | Forward | GAACAGCTTCATCAAGCCCTC |
|  |  | Reverse | GCTTGTGGATGACGAGGTCG |
| TraesCS4A02G059500 | TaITPK2-4A | Forward | CAGAAGTCACTGTCCTCGAC |
|  |  | Reverse | CAGTTGGCGTGGAGTGCAAAC |
| TraesCS4D02G057100 | TaITPK3-4D | Forward | GAGACACACACCCAGAAGTT |
|  |  | Reverse | GACAATCCGCAAGGTCCAG |
| TraesCS1D02G333500 | TaITPK4-1D | Forward | CCATCCCGTGAGGAAGACTT |
|  |  | Reverse | CATCCGTCATTTTGTGGAGG |
| Actin | Actin | Forward | AGGCACCTCTTAACCCTAAAGC |
|  |  | Reverse | GGACAACGGAATCTCTCAGC |
